# Genome-wide analysis of the *CML* gene family and its response to cold stress in *Curcuma alismatifolia*

**DOI:** 10.1186/s12870-025-06898-9

**Published:** 2025-07-12

**Authors:** D. Ying, R. Jintong, W. Yang, F. Zuhong, T. Tian, Z. Hong

**Affiliations:** 1 Guizhou University of Engineering Science, Bijie, China; 2 Guizhou Key Laboratory of Plateau Wetland Conservation and Restoration, Guiyang, China

**Keywords:** *Curcuma alismatifolia*, *CML* genes, Cold tolerance, Transcriptomics, MAPK signaling pathway

## Abstract

Calmodulin-like (CML) transcription factors function as calcium (Ca^2^⁺) signal sensors and play a pivotal role in plant cold resistance. Although this gene family has been identified in various plant species, the characteristics of the *CML* gene family in *Curcuma alismatifolia* and its function under cold stress remain largely unknown. This study identified 202 *CACML* genes in the *Curcuma alismatifolia* genome, which were phylogenetically classified into four clades. Members within each clade shared conserved gene structures and motifs. Gene duplication analysis revealed that segmental duplication events (44 events) served as the primary driving force for the expansion of the *CACML* family. Most homologous gene pairs were subjected to strong purifying selection during evolution, while *CACML86* underwent positive selection in both *C. alismatifolia* and *A. viiosum*. Promoter regions were enriched with *cis*-acting elements associated with growth and development, biotic/abiotic stress responses, and hormone responsiveness, suggesting diverse functional roles for *CACML* genes. Transcriptome analysis indicated that *CACML140* regulates the cold stress response in *Curcuma alismatifolia* via the MAPK cascade signaling pathway. These findings enhance our understanding of CML-mediated cold stress responses in *Curcuma alismatifolia* and establish a framework for the systematic dissection of *CML* gene function and the regulatory network governing cold hardiness.

**Supplementary Information:**

The online version contains supplementary material available at 10.1186/s12870-025-06898-9.

## Introduction

Plants transmit external stimulus signals through messenger networks to internal systems and initiate adaptive responses [1]. Calcium (Ca^2^⁺) acts as an intracellular second messenger, orchestrating plant developmental programs, environmental acclimation, and adaptive responses to diverse abiotic and biotic stresses [2]. When plants encounter diverse environmental challenges-including high temperature, drought, salinity, and pathogen infection fluctuations in cytosolic free calcium ([Ca^2^⁺]cyt) concentrations trigger calcium signaling cascades [3]. These Ca^2^⁺ signals are typically sensed by Ca^2^⁺ sensors or calcium-binding proteins (CBPs), which undergo conformational changes upon Ca^2^⁺ binding, subsequently regulating downstream target genes and propagating Ca^2^⁺ signaling [4]. Distinct calcium-sensing proteins exhibit specialized capacities to decode differential Ca^2^⁺ signatures generated by cytosolic free Ca^2^⁺ [5]. Three major classes of EF-hand domain-containing proteins have been identified as Ca^2^⁺ signal transducers in plants: calmodulin/calmodulin-like proteins (CaMs/CMLs), calcineurin B-like proteins (CBLs), and calcium-dependent protein kinases (CDPKs) [6]. Most calcium sensors utilize the conserved helix-loop-helix structural motif (EF-hand) to mediate Ca^2^⁺ binding. Calmodulin (CaM), one of the most evolutionarily conserved and ubiquitously expressed proteins in eukaryotes, contains four canonical EF-hand domains [7]. In contrast, calmodulin-like proteins (CMLs) are predominantly restricted to plants and select protists, displaying significant variation in EF-hand domain numbers (1–6) [8]. CMLs are characterized by a Dx3D motif, whereas CaMs harbor four calcium-binding DxD motifs. The two α-helices interconnected by the Dx3D motif activate downstream regulatory networks upon calcium ion binding [9].

Calmodulin-like proteins (CMLs) play pivotal roles in plant growth, development, and resistance to biotic/abiotic stresses [10]. In *Arabidopsis thaliana*, 50 CML family members have been identified, with their functions demonstrated to rely on the abscisic acid (ABA)-dependent signaling pathway during abiotic stress responses [11]. Regarding plant development, *AtCML25* and *AtCML36* regulate pollen tube elongation in *A. thaliana*, while cucumber *CML25* promotes fruit expansion by modulating hormone signaling-related genes [12]. Overexpression of wheat *TaCML20* enhances water-soluble carbohydrate accumulation, thereby increasing grain yield [13]. *CMLs* also critically mediate abiotic stress tolerance. For instance, rice *OsCML4* improves drought resistance by reducing reactive oxygen species (ROS) levels [14], whereas tomato *ShCML44* overexpression confers dual tolerance to drought and cold stress [15]. In Medicago truncatula, *MtCML42* interacts with *MtCBF1* and *MtCBF4* to form a regulatory network that upregulates cold stress-responsive genes *MtGolS1* and *MtGolS2*, thereby enhancing freezing tolerance [16]. *Vitis vinifera* L. *CML21* exhibits divergent regulatory patterns under cold stress, underscoring the significance of genetic diversity in environmental adaptation [17]. Despite systematic investigations of CML-mediated stress responses in model plants, the functional characterization and molecular mechanisms of CML family members in *C. alismatifolia* remain poorly understood.

*C. alismatifolia*, a photophilic horticultural ornamental species in the *Zingiberaceae* family [18], is primarily distributed across subtropical and tropical zones. This species demonstrates high ornamental value owing to its vividly colored, morphologically diverse inflorescences and complex aromatic profiles [19]. However, its agricultural cultivation and reproductive development face substantial limitations in cold climatic conditions. Recent genomic advancements have enabled the complete assembly of the *C. alismatifolia* genome, thereby facilitating targeted investigations into Calmodulin-like (*CML*) gene families putatively associated with cold stress response pathways. In this study, we conducted a genome-wide screening to identify *CML* family members in *C. alismatifolia*, followed by systematic characterization of their structural properties, phylogenetic relationships, chromosomal assignments, and conserved domain architectures. To dissect the molecular basis of cold stress adaptation, we further profiled the transcriptional dynamics of select *CML* genes under low-temperature treatments. Through integrative analysis of these multi-dimensional datasets, our work aims to identify pivotal cold-responsive *CML* gene candidates, establishing a robust foundation for downstream functional characterization of *CML*-mediated cold tolerance mechanisms in this economically important ornamental species.

## Materials and methods

### Identification of members, gene physico-chemical properties

Genomic and annotation files of *C. alismatifolia* were downloaded from the NCBI database, while *Arabidopsis* data were obtained from TAIR (https://www.arabidopsis.org/). Candidate *CACML* proteins were identified by BlastP (V = 2.13.0) searching against the *C. alismatifolia* genome using *A. thaliana CML* (*ATCML*) sequences as queries (E-value < 1 × 10⁻^5^). Protein sequences matching the EF-hand domain (PF00036) HMM profile were further retrieved (p-value < 1 × 10⁻^5^) HMMsearch (V = 3.2.0). Overlapping sequences from both methods were validated for EF-hand motif presence using SMART, and only those containing canonical EF-hand motifs were retained as CACML proteins. Biochemical indices of *CACML* proteins, including isoelectric point, molecular weight, and grand average of hydropathicity, were calculated using Expasy tools (https://www.expasy.org/).

### Phylogenetic analysis of *CACML* gene family members

Multiple sequence alignment of *CACML* and *ATCML* protein sequences was performed using Clustal software (V = 3.1). The aligned results were subsequently imported into MEGA (V = 11.0) for phylogenetic tree construction using the Maximum Likelihood (ML) method, with reliability assessed via 1000 bootstrap iterations. Additionally, a phylogenetic tree for CACML proteins was constructed using the same methodology and visualized using iTOL (https://itol.embl.de/).

### Analysis of gene structure, conserved motifs, structural domains and cis-acting element of *CACML* gene family members

Gene structure information of *CACML* genes was extracted from the *C. alismatifolia* genome annotation file, with positional data for CDS and UTR regions statistically analyzed. Gene structure visualization was performed using TBtools software (https://github.com/CJ-Chen/TBtools-II) [20]. *CACML* gene sequences were uploaded to the MEME (http://memeesuite.org/tools/meme) and InterPro database (https://www.ebi.ac.uk/interpro/), with the motif number set to 10 for analysis. The 2000 bp upstream sequences of *CACML* genes were extracted as promoter regions, and cis-acting elements were identified using the PlantCARE database (http://bioinformatics.psb.ugent.be/webtools/plantcare/html) and classified into functional categories.

### Chromosomal location and duplication events analysis of *CACML* gene

Gene duplication events of *CACML* genes were analyzed using MCScanX software (http://chibba.pgml.uga.edu/mcscan2/) [21], with visualization performed in Circos. Collinear relationships of *CML* genes between *C. alismatifolia*, *A. viiosum*, and *A. tsaoko* were analyzed by MCScanX, and the results were visualized using TBtools software. Collinear *CML* gene pairs were statistically identified, and their Ka/Ks values were calculated using KaKs_Calculator (V = 3.0).

### Tissue expression analysis of *CACML* gene family members

C. *alismatifolia* were cultivated in the greenhouse of Guizhou University of Engineering Science (27°17′34.436″ N, 105°18′46.069″ E). For cold stress treatment, *C*. *alismatifolia* were transferred into two separate growth chambers maintained at 4 °C and 25 °C respectively. After 12 h of treatment, leaf samples from three biological replicates per treatment were collected for total RNA extraction. Sequencing libraries were constructed using 1 μg of RNA with the NEBNext Ultra RNA Library Prep Kit (NEB, Ipswich) for Illumina. Sequencing reads were aligned to the *C. alismatifolia* genome, and transcript assembly and FPKM value calculation were performed using the StringTie program (v. = 2.1.1) with default parameters. Differentially expressed mRNAs were identified using the DESeq2 package (v. = 1.33.5) in R software with default settings, where genes with |fold change|> 2 and *P*-value < 0.05 were defined as significantly altered.

KEGG pathway enrichment analysis was conducted for differentially expressed genes (DEGs) meeting |fold change|> 2 and *P* < 0.05. Corrplot R package was performed to calculate gene correlations (R^2^ > 0.9). Volcano plots, bubble enrichment maps, correlation heatmaps, and cluster heatmaps were generated using ggplot2 R package (V = 2.1).

### Quantitative reverse transcription-polymerase chain reaction (qRT-PCR) Analysis

First-strand cDNA was synthesized from total RNA of samples using the HiScript®II cDNA Synthesis Kit. Primers were designed using NCBI primer blast(https://www.ncbi.nlm.nih.gov/tools/primer-blast/) with an amplified fragment length of 150–250 bp (Supplementary Table 2). The iTaq Universal SYBR®Green Supermix (Tiangen Biotech, Beijing) was used in a 20 µL reaction volume, with three biological replicates set for each sample. *ACT2* expression level was used as an internal reference. Gene expression levels were detected on a Biosystems 7500 Real-Time PCR system (Thermo Fisher Scientific, Waltham), and expression differences were calculated using the 2^−ΔΔCT^ method.

### Statistical analysis

Statistical analyses and figure preparation were performed using GraphPad Prism 8.0 and Adobe Illustrator 2020.

## Results

*A. thaliana AtCML* protein sequences were selected to conduct BLASTP searches against the Curcuma alismatifolia genome. Candidate *CML* protein sequences were further filtered based on the EF-hand domain (PF00036), a structural signature characteristic of *CML* proteins. In this study, 202 *CML* genes were identified from the *C. alismatifolia* genome and systematically named *CACML1*-*CACML202* according to their chromosomal positions (Supplementary Table 1). Sequence analysis revealed that all *CACML* proteins harbor a conserved EF-hand domain. The *CML* gene family displayed remarkable structural diversity, with protein lengths spanning 73–1447 amino acids (*CACML134*-*CACML143*). Molecular weight calculations showed values of 8.24–164.18 kDa (*CACML134*-*CACML143*), while isoelectric points (PI) ranged 3.64 (*CACML125*)−9.77 (*CACML*176). 29 CACML proteins were classified as basic proteins (PI > 7).

**Fig. 1:**
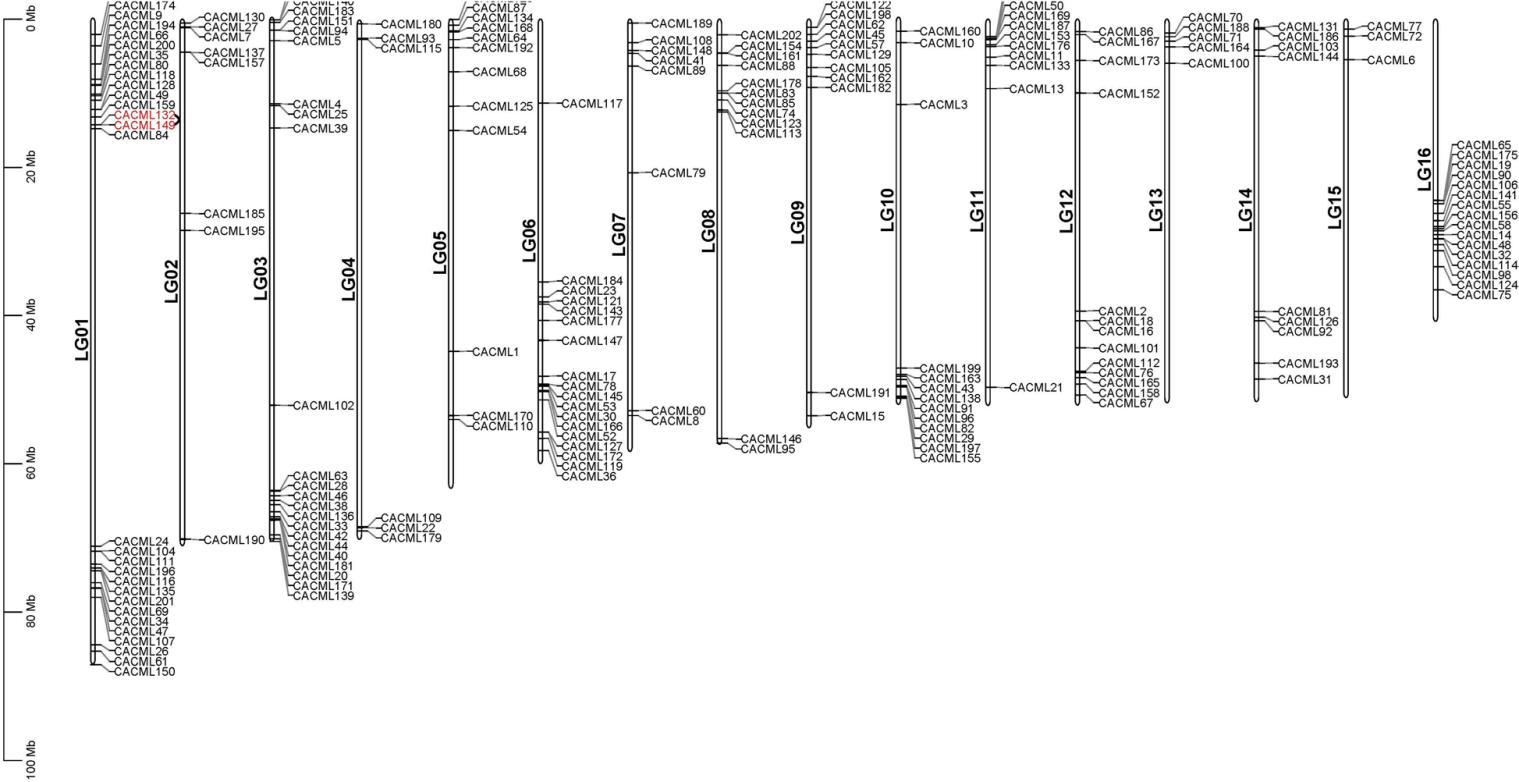
Chromosomal localization of *CACML* genes in *C. alismatifolia*. Note: Chromosome sizes are indicated by the leftmost vertical scale (Mb); chromosome numbers are positioned on the left side of each bar; gene pairs highlighted in red represent tandem duplicate pairs

Chromosomal mapping of the 202 *CACML* genes revealed that 197 members were chromosomally localized (Fig. 1), with 5 genes remaining unassigned due to incomplete genome annotation. The 197 *CACML* genes exhibited non-uniform distribution across 16 chromosomes, with chromosome 1 harboring the highest number (29) and chromosome 15 the lowest (3). only a single tandem duplicate pair was identified within the *CACML* gene family.

**Fig. 2:**
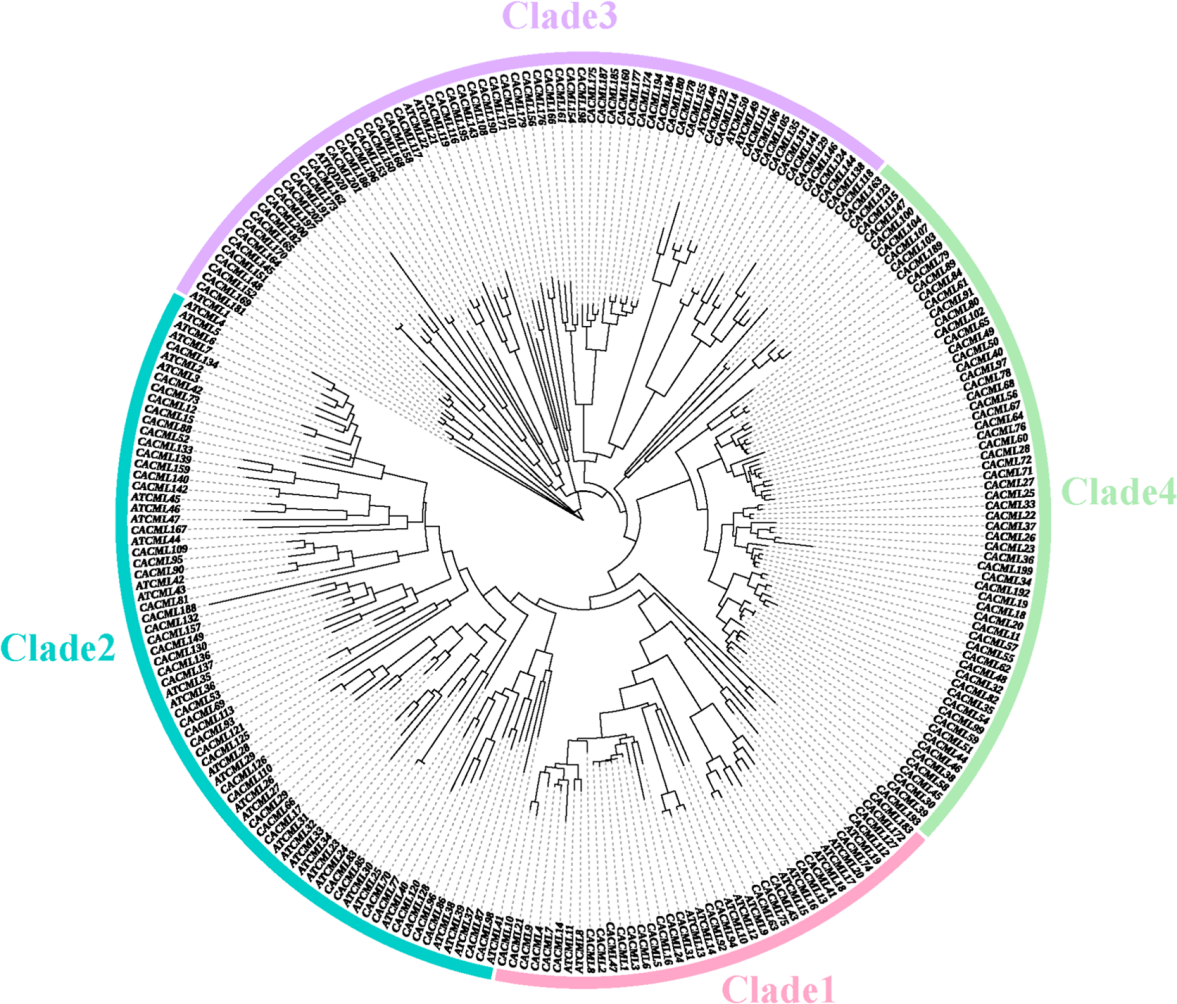
Phylogenetic tree of the *CML* gene family in *C. alismatifolia* and *A. thaliana*. Note: The tree was constructed using the Maximum likelihood (ML) method with 1000 bootstrap replicates

To explore the evolutionary relationships and functional divergence of the *CACML* gene family, a phylogenetic tree was constructed using 202 *CACML* sequences and 50 *ATCML* (Fig. 2). Phylogenetic analysis grouped the *CACML* genes into four distinct clades (Clade 1–4), with Clade 3 containing the largest number of members (83) and Clade 1 the smallest (26). Notably, Clade 4 lacked *A. thaliana*, suggesting potential species-specific functional innovations within this Clade.

To investigate the evolutionary relationships and functional divergence of the *CACML* gene family, a phylogenetic tree was constructed using 202 *CACML* sequences and 50 *AtCML* sequences from *A. thaliana* (Fig. 2). Phylogenetic analysis classified *CACML* genes into four distinct clades (Clade 1–4), each containing *CACML* members. Clade 3 exhibited the largest membership (83 genes), while Clade 1 contained the fewest (26 genes). Clade 4 lacked *A. thaliana* homologs, suggesting potential species-specific functional divergence within this clade. High sequence similarity observed among CML proteins from diverse plant species implies conserved functional roles and shared evolutionary trajectories across clade.

**Fig. 3:**
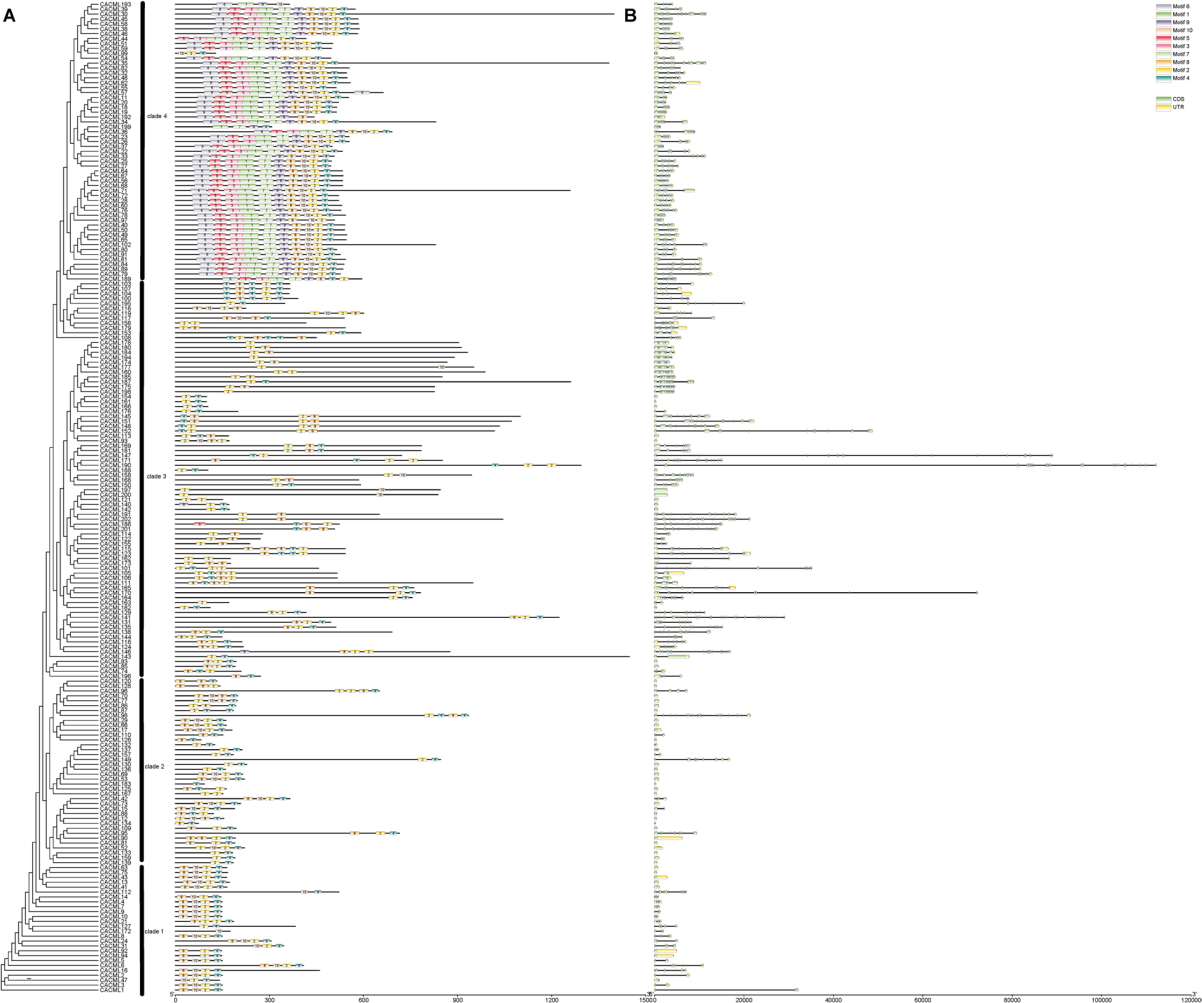
Conserved motifs and gene structure analysis of 202 *CACML* genes. Note: **a** Schematic diagram of conserved motifs in *CACML* protein sequences, where different colored boxes (1–10) represent distinct conserved motifs. **b** Exon–intron-untranslated region (UTR) structure of *CACML* genes, with exons shown as pink boxes, introns as black lines, and UTRs as green boxes

Previous studies have demonstrated correlations between gene structural features, expression patterns, and functional divergence [22]. Using MEME, we identified 10 conserved motifs among the 202 *CACML* genes (Fig. 3A). Motifs 2 and 4 corresponded to EF-hand domains, which were universally conserved across all *CACML* proteins. All 10 motifs were retained in Clade 4 members, whereas most *CACML* proteins in Clades 1–3 comprised Motifs 2, 4, 8, and 10. The shared motif composition among phylogenetically closely related protein family members suggests potential functional differentiation between *CACML* proteins in Clades 1–3 and Clade 4. These findings indicate that conserved motif organization may underpin functional coherence within phylogenetic clade.

To further elucidate structural variations in *CACML* genes, we analyzed their conserved motifs, exon–intron architectures, and untranslated regions (UTRs). Results revealed that most *CACML* genes possess two UTRs and multiple exons. Specifically, members of Clade 1 and Clade 4 exhibited relatively fewer exons (0–16) (Fig. 3 B). In contrast, Clade 2 and Clade 3 displayed more complex gene structures, with *CACML141* in Clade 3 containing the highest number of exons (34), indicative of evolutionary diversification. The simpler gene architectures of Clade 1 and Clade 4 members suggest their potential for rapid transcriptional activation due to reduced intron splicing steps, thereby shortening stress response latency. Structural divergence of *CACML* genes across clades may be associated with lineage-specific functional specialization.

**Fig. 4:**
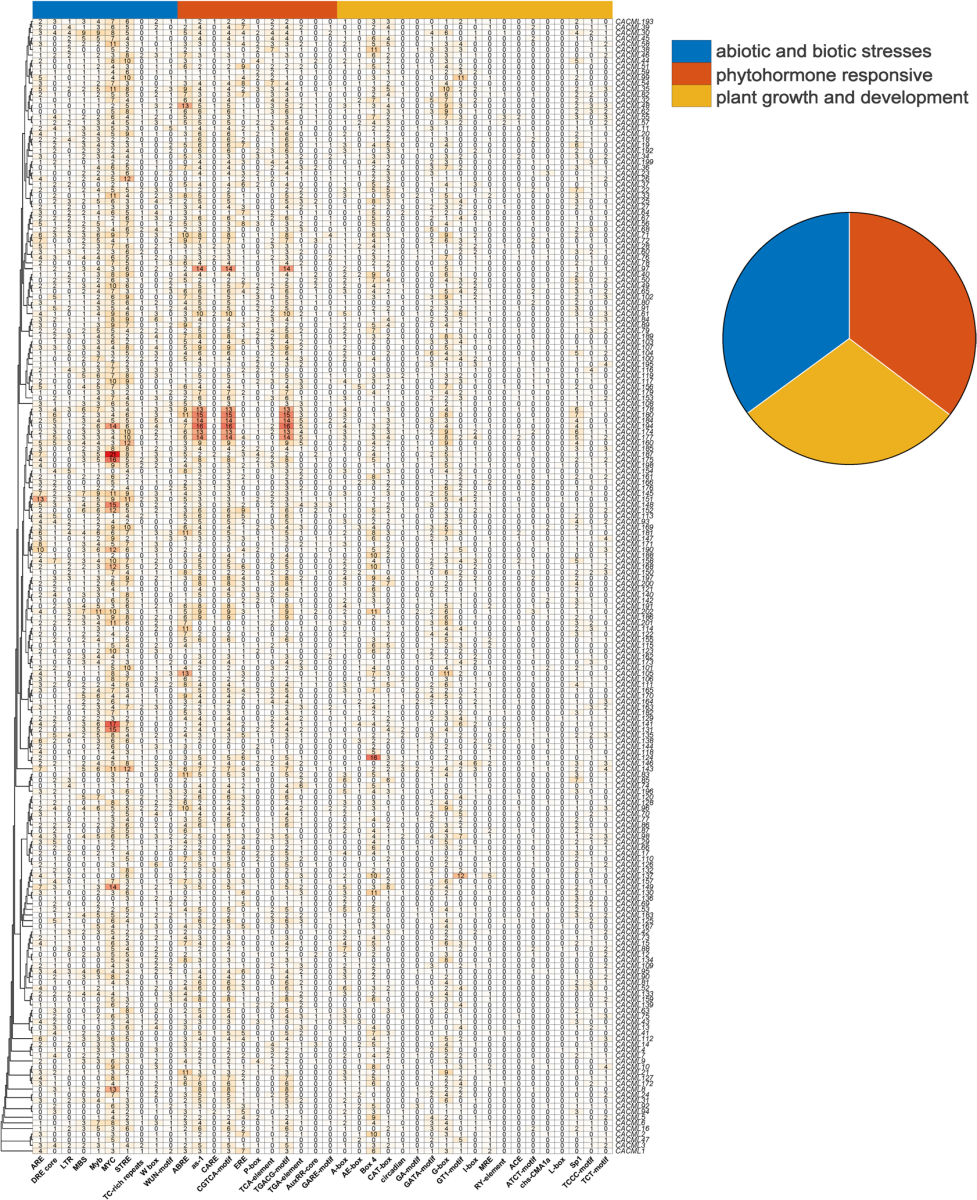
Cis-elements in the *CACML* promoters. Note: The vertical axis represents *CACML* genes, while the horizontal axis shows different types of cis-acting elements. The text annotations denote the number of corresponding cis-acting elements, with red color indicating higher abundance

**Fig. 5:**
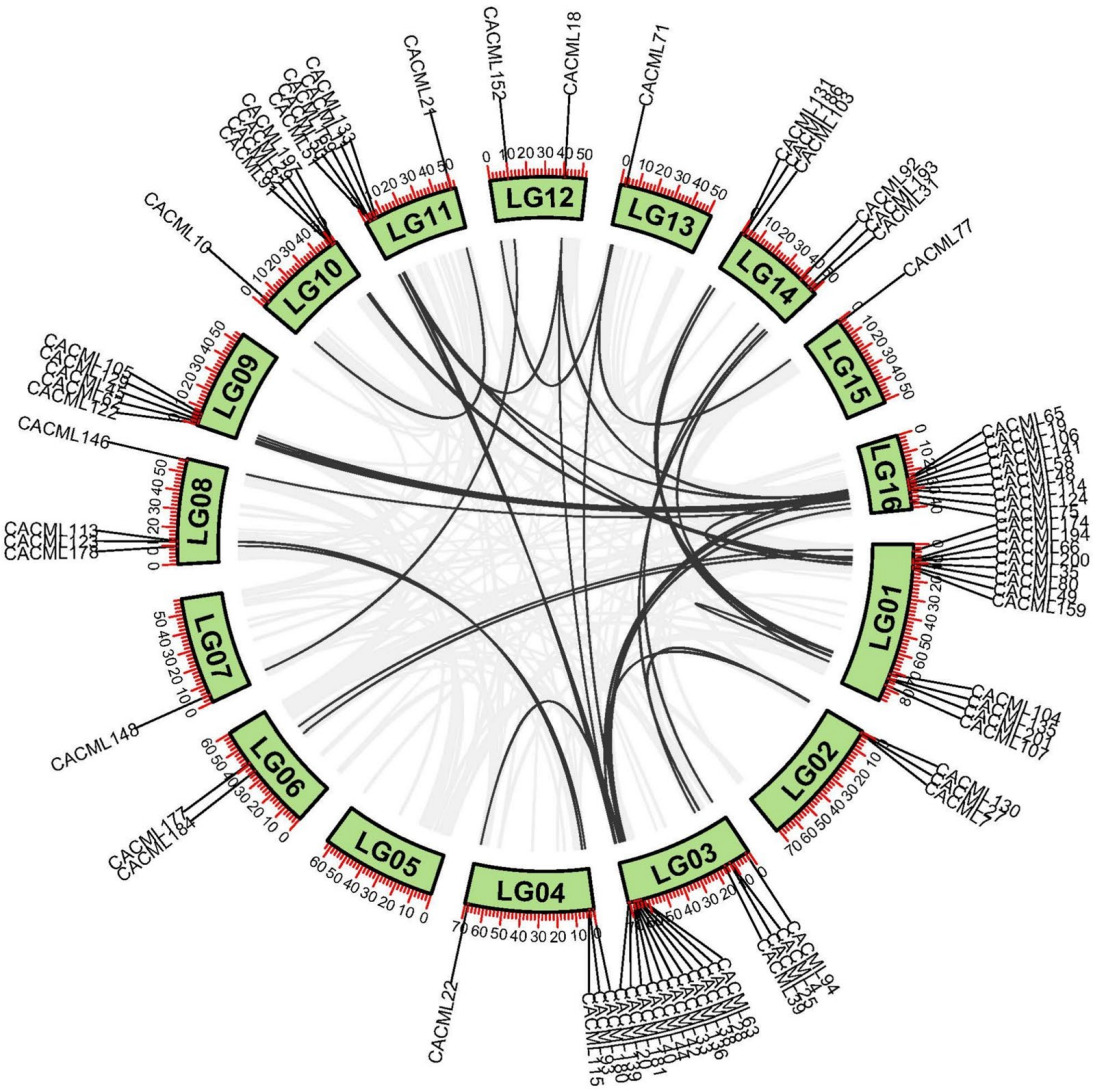
Intraspecies homology analysis of *CACML* genes. Note: Black lines represent duplicated *CACML* gene pairs, while gray lines indicate whole genome duplicated gene pairs

Promoter analysis of the 2000 bp upstream regulatory regions of 202 *CACML* genes identified abiotic/biotic stress-responsive elements, phytohormone-responsive elements, and growth/development-related cis-elements (Fig. 4, A,B). Stress-responsive and phytohormone-related elements were the most abundant, with MYC elements involved in cold response showing the highest frequency (1094) (Fig. 4, A). Notably, *CACML187* contained the largest number of MYC cis-acting elements (21) (Fig. 4, A). Among growth/developmental elements, Box4 (644 occurrences) was most prevalent, associated with light signal perception and transduction. Within hormone-responsive elements, jasmonic acid-related CGTCA-motif (829 instances) dominated (Fig. 4, A). These findings collectively indicate that the *CACML* gene family plays regulatory roles in turmeric's responses to diverse abiotic/biotic stresses and growth/developmental processes (Fig. 5).

To investigate the evolutionary driving forces underlying the expansion of the *CACML* family, we performed genome-wide collinearity analysis on 202 *CACML*. A single tandem duplication event (*CACML132*-*CACML149*) was identified. Furthermore, 44 segmental duplication events involving 83 *CACML* genes were detected. The predominance of segmental duplication events over tandem duplications suggests that segmental duplication served as the primary evolutionary mechanism driving *CACML* gene family expansion in *C. alismatifolia*.

**Fig. 6:**
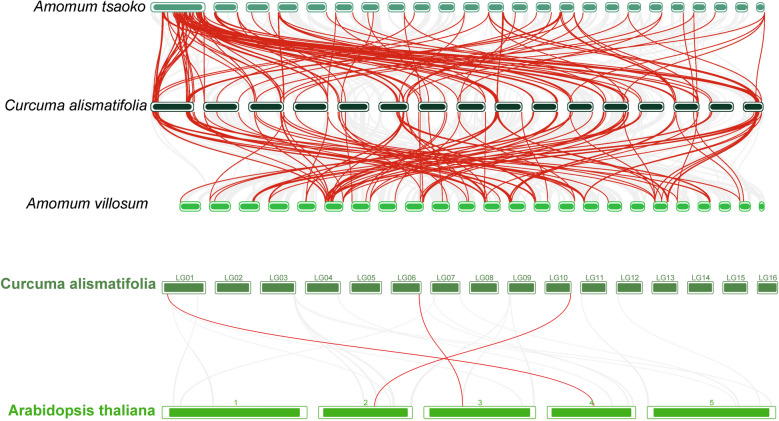
Collinear *CML* genes between *C. alismatifolia* and *A. viiosum*, and between *C. alismatifolia* and *A. tsaoko*, *C. alismatifolia* and *A. thaliana* are shown. Red lines represent collinear gene pairs

To further characterize the evolutionary trajectory of *CML* genes in *C. alismatifolia*, interspecific collinearity analysis was performed among three *Zingiberaceae* species (*C. alismatifolia*, *Amomum viiosum*, and *Amomum tsaoko*) and *A. thaliana*. A total of 161 orthologous pairs were identified between *C. alismatifolia* and *A. tsaoko*, with 148 orthologs detected between *C. alismatifolia* and *A. viiosum* (Fig. 6). These orthologous relationships primarily exhibited one-to-many or many-to-many collinear patterns, e.g., *CACML20* shared two orthologs in both *A. tsaoko* and *C. alismatifolia*, indicating potential functional redundancy. Notably, no collinearity was observed between *A. tsaoko*/*C. alismatifolia* genes and the previously identified tandem duplication pair, suggesting that this tandem duplication event underwent independent evolution in the *C. alismatifolia* genome.

To further characterize the evolutionary trajectory of CML genes in *C. alismatifolia*, interspecific collinearity analysis was performed among three *Zingiberaceae* species (*C. alismatifolia*, *A. viiosum*, and *A. tsaoko*) and A. thaliana. A total of 161 orthologous pairs were identified between *C. alismatifolia* and *A. tsaoko*, with 148 orthologs detected between *C. alismatifolia* and *A. viiosum* (Fig. 6). These orthologous relationships primarily exhibited one-to-many or many-to-many collinear patterns, e.g., *CACML20* shared two orthologs in both *A. tsaoko* and *C. alismatifolia*, indicating potential functional redundancy. Notably, no collinearity was observed between *A. tsaoko*/*C. alismatifolia* genes and the previously identified tandem duplication pair, suggesting that this tandem duplication event underwent independent evolution in the *C. alismatifolia* genome. Only three pairs of orthologous CML genes exist between *C. alismatifolia* and Arabidopsis thaliana (*CACML118*-*ATCML49*, *CACML127*-*ATCML11*, *CACML155*-*ATCML48*). These three *CACML* genes also exhibit synteny with *A. viiosum* and *A. tsaoko*. This interspecies synteny indicates that these three *CACML* genes have been conserved during evolution.

Ka/Ks ratio analysis revealed that the majority of *Zingiberaceae CML* genes have been subjected to purifying selection (Ka/Ks < 1) (Supplementary Table 3). A notable exception was *CACML86*, which showed contrasting evolutionary pressures, its ortholog in *A. viiosum* experienced positive selection (Ka/Ks > 1), while the *A. tsaoko* paralog remained under purifying selection (Ka/Ks < 1).

Ka/Ks ratio analysis indicates that the *CML* genes in *C. alismatifolia* and *A. thaliana*, as well as most *CML* genes in *Zingiberaceae*, underwent purifying selection (Ka/Ks < 1) (Supplementary Table 3). A notable exception was *CACML86*, which showed contrasting evolutionary pressures, its ortholog in *A. viiosum* experienced positive selection (Ka/Ks > 1), while the *A. tsaoko* paralog remained under purifying selection (Ka/Ks < 1).

**Fig. 7:**
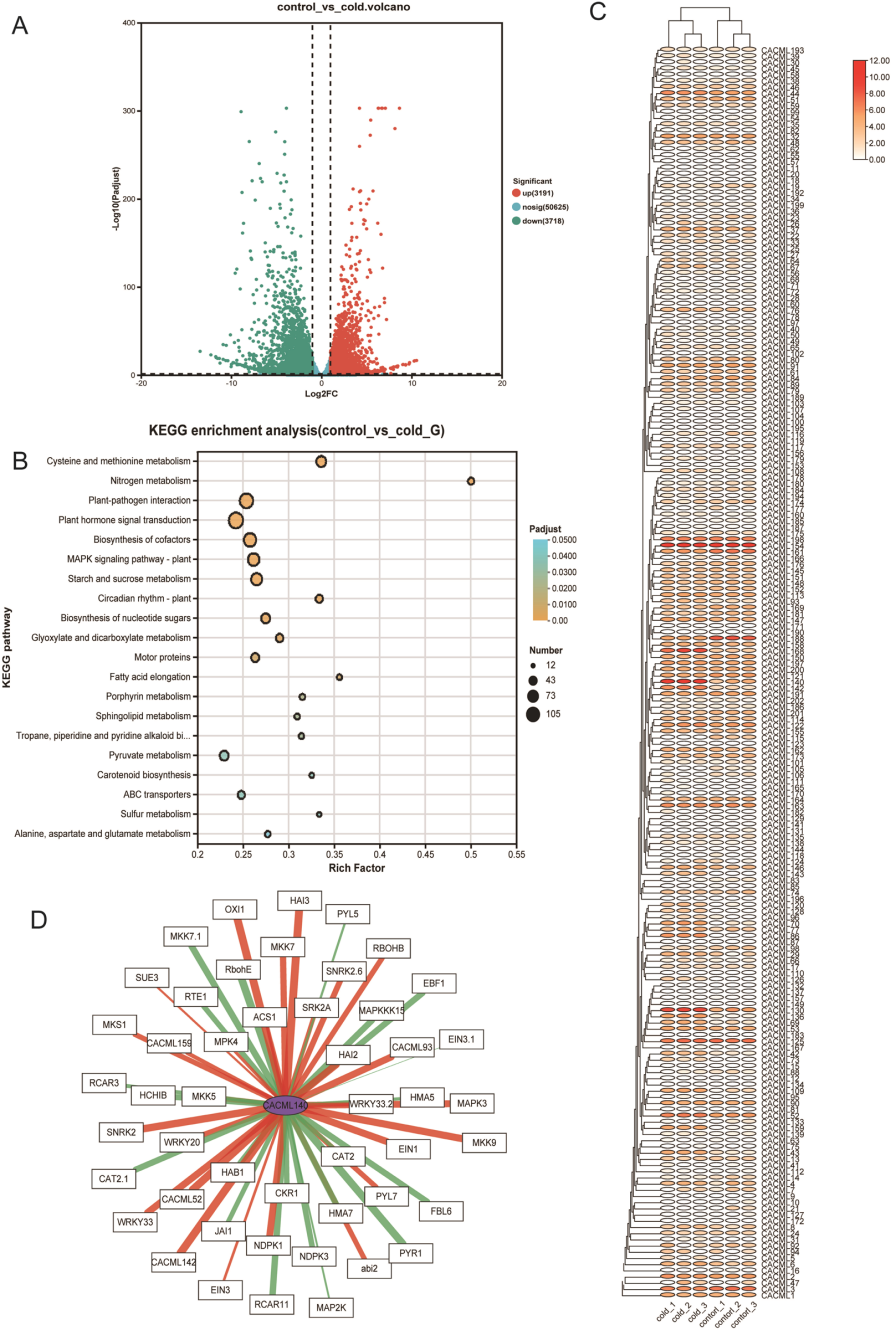
Cold-resistant transcriptome analysis of *C. alismatifolia*. Note: **A** Volcano plot of differentially expressed genes in the cold-resistant transcriptome. **B** KEGG enrichment bubble map of differentially expressed genes in the cold-resistant transcriptome. **C** Heatmap of *CACML* gene expression levels in the cold-resistant transcriptome. **D** Correlation diagram between MAPK signaling pathway-enriched genes and CACML140

To further investigate the potential molecular mechanisms of *CACML* gene involvement in cold stress response in *C. alismatifolia*, six samples were selected for transcriptome analysis. Six cDNA libraries were constructed, with post-filtering data showing Q20 and Q30 values both exceeding 95%, and over 90% of clean reads successfully mapped to the *C. alismatifolia* genome. Differentially expressed genes (DEGs) were identified and visualized through volcano plots. Compared with the control group, 6,909 DEGs (3,191 upregulated and 3,718 downregulated) were detected in cold-treated samples (Fig. 7A). KEGG pathway enrichment analysis of these 6,909 DEGs revealed the top 10 significantly enriched pathways based on Padjust values. The results demonstrated significant enrichment of DEGs in the MAPK signaling pathway, plant hormone signal transduction, and nitrogen metabolism (Fig. 7B).

Expression profiling of *CACML* genes in this dataset (Fig. 7, C) identified *CACML140* as a significantly upregulated gene enriched in the MAPK signaling pathway. Co-expression analysis within this pathway revealed positive correlations between *CACML140* and *MKK7*/*MKK9,* whereas *RCAR11* and *MKK7* showed negative correlations (Fig. 7, D). Collectively, these transcriptomic data suggest that *CACML140* modulates the MAPK signaling cascade during cold stress in *C. alismatifolia*.

**Fig. 8:**
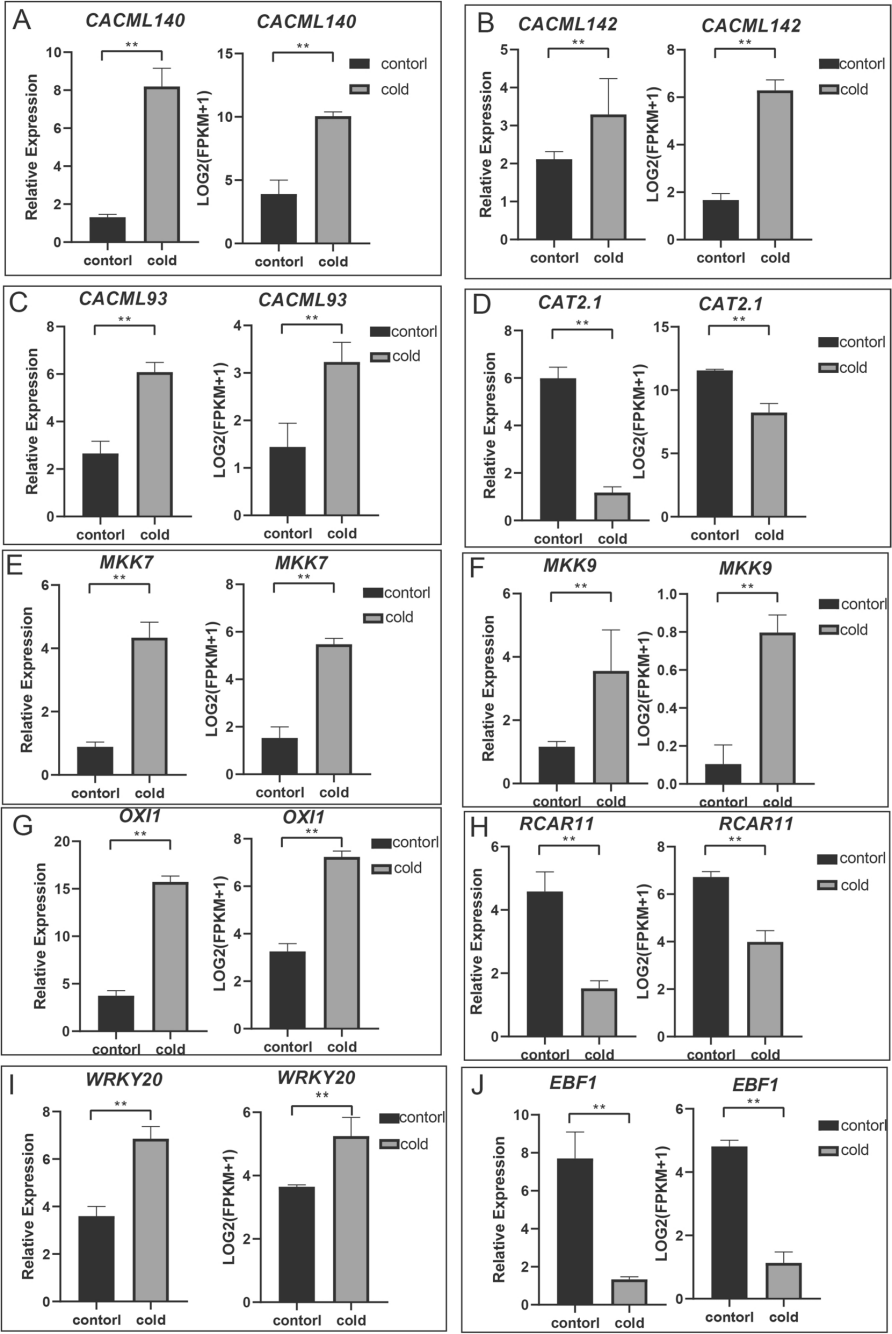
Relative expression of genes related to the MAPK signaling pathway in response to cold treatment. Error bars represent standard error, ** denote highly significant differences (*p* < 0.01)

To validate RNA-seq findings, qRT-PCR analysis was performed to characterize the expression profiles of *CACML140* and MAPK signaling components (*MKK7*, *MKK9*, and *RCAR11*) in leaves subjected to 4 °C cold treatment for 12 h (Fig. 8). Statistical analysis revealed significant upregulation of *CACML140*, *CACML142, CACML93, MKK7*, *OXI1*, *WRKY20* and *MKK9*, whereas *RCAR11, EBF1, CAT2.1* showed marked downregulation under cold stress. These results suggest that C*ACML140* and the MAPK signaling cascade play critical roles in cold acclimation of *C. alismatifolia*.

## Discussion

Calmodulin-like proteins (*CMLs*), ubiquitous calcium-binding sensors in eukaryotes, regulate plant growth, development, and responses to biotic and abiotic stress signals [23]. In this study, 202 *CACML* genes were identified in *C. alismatifolia*. Each *CACML* gene possesses at least one EF-hand motif, conforming to the conserved structural characteristics of typical calmodulin-like proteins. With the increasing availability of high-quality plant genomes, the *CML* gene family has been systematically identified at the whole-genome level, revealing considerable variation in gene number across species: *Glycine max* (41 *GmCMLs*) [24], common bean (111 *PvCMLs*) [25], and peanut (191 *AhCMLs*) [26]. The number of *CML* genes may depend on selective pressures and functional demands encountered during plant evolution [27]. Given that most *CACML* genes also contain introns in their structure, it is speculated that *CACMLs* may have undergone distinct evolutionary pressures to facilitate adaptation and function in diverse ecological niches inhabited by *C. alismatifolia*.

Chromosomal localization and subcellular prediction analyses revealed that 197 *CACML* proteins are unevenly distributed across *C. alismatifolia* chromosomes, with chromosome 1 harboring the highest number. This distribution pattern aligns with previously reported *CML* gene distributions in other plant species [28]. Phylogenetic analysis using *A. thaliana CML* genes classified *CACML* genes into four distinct clades (Clade 1–4). Notably, Clade 4 lacked *A. thaliana* orthologs, indicating that dicotyledonous *CML* genes underwent functional diversification during evolution to adapt to environmental selection pressures and acquire novel functions. All four clades retained motif 2 and 4(Dx3D domain), with Clade 4 uniquely containing all 10 conserved motifs. This expanded motif repertoire likely contributes to specialized protein functions within Clade 4.

Tandem duplication and segmental duplication are major factors driving the expansion of gene families [29]. Genes arising from these duplication events frequently undergo subsequent sequence and functional divergence, enabling plants to adapt to diverse environmental challenges [30]. In *C. alismatifolia*, 44 segmental duplication events and one tandem duplication pair (*CACML132*-*CACML149*) were identified. As observed in other monocots, segmental duplication serves as the primary driver for CML gene family expansion in monocotyledonous plants.

Inter-species synteny analysis between *C. alismatifolia* and *A. tsaoko*, *A. viiosum*, and the eudicot model *A. thalian*a revealed distinct evolutionary patterns. Complex homologous relationships were observed with *A. tsaoko* and *A. viiosum*, yielding 161 and 148 syntenic gene pairs, respectively. In contrast, only three syntenic gene pairs were identified with *A. thaliana*, likely reflecting the distant evolutionary divergence between monocots and eudicots. tandem duplications in *C. alismatifolia* exhibited no syntenic relationship with those in *A. tsaoko* or *A. viiosum*, indicating independent evolution of these tandemly duplicated genes with potential species-specific functional innovations. Synteny of *ATCML11* was further detected in other eudicots (*Brassica oleracea *[31] and *Phoebe bournei *[32]), demonstrating its ancestral conservation across monocot and eudicot lineages. The widespread occurrence of one-to-many or many-to-many homologies (e.g., *CACML20* sharing two homologs in both *A. viiosum* and *A. tsaoko*) suggests these genes arose either from duplication events in a common ancestor or through independent convergent evolution. The conservation of such homologous genes may underpin essential biological functions conserved across *Zingiberaceae* species, including growth, development, and stress responses.

Positive selection (Ka/Ks > 1) drives adaptive evolution in response to specific environmental pressures or ecological interactions. Conversely, purifying selection (Ka/Ks < 1) indicates conserved functional constraints [33]. The majority of *CACML* genes in *C. alismatifolia* underwent purifying selection (Ka/Ks < 1), demonstrating strong functional constraints. This evolutionary pattern purging deleterious mutations and preserving gene function integrity suggests their involvement in fundamental biological processes essential for plant survival and reproduction [34]. CML genes in plants such as *Passiflora edulis *[35] and *Ralstonia solanacearum* [26] consistently exhibit purifying selection. In contrast, *CACML86* diverged from other *CACML* genes, experiencing positive selection in both *C. alismatifolia* and *A. viiosum*. Shared ecological niches or evolutionary histories may underlie this convergent adaptive trajectory in *CACML86* between the two species.

Cis-acting element analysis of 2000 bp upstream promoter regions revealed diverse regulatory motifs in *CACML* genes. Abundant abiotic and biotic stresses and phytohormone responsive cis-acting elements suggest multifunctional roles in environmental adaptation and hormonal signaling. The prevalence of MYC cis-acting elements (1094), particularly in *CACML187* (21), highlights its potential as a key regulator in cold stress response pathways [36]. These elements likely activate downstream cold tolerance genes through MYC-mediated transcriptional regulation. Jasmonic acid responsive CGTCA-motifs (829) dominate hormone-related elements, indicating strong regulation by jasmonic acid signaling [37]. This suggests *CACML* genes coordinate plant defense responses against herbivores, pathogens, and developmental processes involving jasmonic acid [38].

Drought, cold, and other abiotic stresses significantly impact the growth and development of *C. alismatifolia *[39]. Understanding the gene regulatory network underlying its response to cold stress is crucial for its development [40]. Cold stress induces dynamic changes in intracellular calcium ion (Ca^2^⁺) concentration in plants. Calmodulin-like proteins (CMLs) act as calcium sensors [41]. They bind Ca^2^⁺ ions to form Ca^2^⁺-CML complexes, which interact with downstream target proteins [42]. These interactions modulate the expression of cold-responsive genes, thereby initiating the plant's cold stress response. *AtCML9*, *18*, *24*, and *37* are all involved in responses to abiotic stress in *A. thaliana *[43]. Soybean *GsCML27* is induced by salt stress, and its heterologous expression enhances salt tolerance [44]. Tomato *ShCML44* is induced by multiple abiotic stresses, and overexpression of *ShCML44* improves tolerance to both cold and salt stress [45]. Collectively, these findings indicate that *CML* genes function as key regulators enhancing plant resistance to abiotic stress. In this study, transcriptomic analysis of *C. alismatifolia* under cold stress identified 6,909 DEGs. KEGG pathway enrichment analysis revealed significant enrichment in the MAPK signaling pathway and nitrogen metabolism. The plant MAPK signaling pathway is associated with signal transduction under cold stress. For instance, the Arabidopsis *AtCRLK1*-*AtMEKK1/2*-*AtMPK4/6* cascade enhances cold tolerance by antagonizing the *AtMPK3/6* pathway [46], while *Medicago truncatula MtCTLK1* or *Medicago falcata MfCTLK1* influences cold resistance via the CBF transcriptional cascade, antioxidant defense, and proline accumulation [47]. *CACML140* exhibited the most significant differential expression and was enriched within the MAPK cascade signaling pathway. The above results indicate that *CACML140* regulates the cold tolerance of *C. alismatifolia* through the MAPK cascade signaling pathway, a conclusion consistent with validation by qRT-PCR.

## Conclusion

This study identified 202 CML genes in *C. alismatifolia*, all containing EF-hand domains. Based on phylogenetic relationships, they were classified into four distinct clades. Clade 4 contained the complete motif composition, and genes within the same subgroup exhibited similar exon–intron structures. Segmental duplication events were identified as the primary driver for the expansion of the *CACML* gene family. Most genes underwent purifying selection (Ka/Ks < 1). *CACML86* experienced positive selection in both *C. alismatifolia* and *A. viiosum*, suggesting a potential shared evolutionary history between these species. Promoter analysis revealed that *CACML* promoters are enriched in cis-elements responsive to abiotic/biotic stress, plant hormones, and growth/development. RNA-Seq and qRT-PCR results indicated that *C. alismatifolia* may respond to cold stress via *CACML140*, potentially through the MAPK cascade signaling pathway. This research provides a theoretical foundation for deciphering the cold stress regulatory network in *C. alismatifolia*, particularly elucidating the regulatory role of the *CACML* gene family. The findings not only enrich the understanding of the evolutionary history and functional studies of plant *CML* gene families but also offer valuable candidate gene resources for the future development of cold-tolerant germplasm.

## Supplementary Information

Supplementary Material 1.

Supplementary Material 2.

Supplementary Material 3.

## Data Availability

The cleaned raw transcriptome reads have been deposited in the National Center for Biotechnology Information (NCBI) Sequence Read Archive (SRA) under BioProject accession number PRJNA1212824 (available at: https://www.ncbi.nlm.nih.gov/bioproject/PRJNA1212824). All data generated or analyzed during this study are included in this published article and its supplementary information files.

## Abbreviations

CML: Calmodulin-like protein
CaM: Calmodulin protein
CBL: Calcineurin B-like protein
CDPK: Calcium-dependent protein kinases
ABA: Abscisic Acid
NJ: Neighbor-Joining
FPKM: Fragments Per Kilobase of transcript per Million mapped reads
qRT-PCR: Quantitative Reverse Transcription-Polymerase Chain Reaction
SYBR: Synthetic Green
ACT2: Actin 2
PI: Isoelectric Point
KEGG: Kyoto Encyclopedia of Genes and Genomes
DEGs: Differentially Expressed Genes
MAPK: Mitogen-Activated Protein Kinase
